# Neural Mechanisms of Circadian Regulation of Natural and Drug Reward

**DOI:** 10.1155/2017/5720842

**Published:** 2017-11-21

**Authors:** Lauren M. DePoy, Colleen A. McClung, Ryan W. Logan

**Affiliations:** ^1^Department of Psychiatry, University of Pittsburgh School of Medicine, Pittsburgh, PA, USA; ^2^Translational Neuroscience Program, Department of Psychiatry, University of Pittsburgh School of Medicine, Pittsburgh, PA, USA; ^3^Center for Neuroscience, University of Pittsburgh, Pittsburgh, PA, USA; ^4^The Jackson Laboratory, Bar Harbor, ME, USA

## Abstract

Circadian rhythms are endogenously generated near 24-hour variations of physiological and behavioral functions. In humans, disruptions to the circadian system are associated with negative health outcomes, including metabolic, immune, and psychiatric diseases, such as addiction. Animal models suggest bidirectional relationships between the circadian system and drugs of abuse, whereby desynchrony, misalignment, or disruption may promote vulnerability to drug use and the transition to addiction, while exposure to drugs of abuse may entrain, disrupt, or perturb the circadian timing system. Recent evidence suggests natural (i.e., food) and drug rewards may influence overlapping neural circuitry, and the circadian system may modulate the physiological and behavioral responses to these stimuli. Environmental disruptions, such as shifting schedules or shorter/longer days, influence food and drug intake, and certain mutations of circadian genes that control cellular rhythms are associated with altered behavioral reward. We highlight the more recent findings associating circadian rhythms to reward function, linking environmental and genetic evidence to natural and drug reward and related neural circuitry.

## 1. Introduction

Circadian rhythms are near 24-hour oscillations in physiology and behavior, which help an organism adapt to changes in their internal or external environment to optimize function and promote survival. Synchrony or temporal coordination of circadian oscillators between central and peripheral tissues, in addition to alignment of those oscillators with the external environment, is important for maintaining organismal homeostasis. For example, disruptions to the circadian system are associated with poor health outcomes, including neurological diseases and psychiatric disorders. The circadian system is hierarchically organized in mammals, with the master circadian pacemaker of the suprachiasmatic nucleus (SCN) receiving photic (i.e., light) input from the eye and nonphotic inputs from disparate brain regions. The SCN relays timing information to other areas of the brain and the periphery through neural and humoral outputs. Coordination of these systems by the SCN is important for modulating the phases of peripheral oscillators. At the cellular level, timing information is kept by the molecular clock, a series of transcriptional and translational negative feedback loops. These feedback loops are driven primarily by circadian genes and their proteins. The transcription of *Period* (*Per1*, *Per2*, and *Per3*) and *cryptochrome* (*Cry1* and *Cry2*) genes are driven by CLOCK and BMAL1 heterodimers binding to enhancer elements (E-boxes) at their promoters. The accumulation of PERs and CRYs in the cytoplasm eventually translocate to the nucleus to interaction with CLOCK/BMAL1, effectively repressing their own transcription. Other genes form “auxiliary” loops providing stability and robustness to the core molecular clock loops.

Disruptions in sleep and circadian rhythms are observed across many psychiatric disorders (reviewed in [1]; reviewed in [2]), including addiction (reviewed in [3, 4]; reviewed in [5–7]). Moreover, polymorphisms in specific circadian genes are associated with drug intake and dependence in humans [8–12]. Evidence from animal studies suggests a bidirectional relationship between circadian rhythms and drugs of abuse. While circadian disruption, whether environmental or genetic, can impact “reward” function and drug-related behaviors, such as self-administration [13–16], exposure to drugs of abuse can also alter molecular, cellular, and behavioral rhythms [16–19], the expression of circadian genes [20], and sleep [21, 22].

After prolonged consistently timed exposure to drugs of abuse, rhythms may become entrained, as evident from anticipatory behavioral activity paralleled by temporal-specific changes of molecular rhythms in “reward” circuits [23–25]. Restricted feeding schedules may also entrain locomotor rhythms where anticipatory activity precedes feeding (reviewed in [26]), suggesting food, possibly acting a “natural reward,” may be controlled or impacted by the circadian system. Natural and drug rewards seem to impact similar neural circuits (e.g., mesocorticolimbic dopamine system) with consequences on reward-related behaviors, such as behavioral anticipation. This review will examine the role of circadian rhythms in natural and drug reward, particularly the overlapping circuit, cellular, and molecular mechanisms of food and drug response. We also discuss the parallels between human and animal research within circadian rhythms and addiction, while highlighting key areas of future investigation.

## 2. Natural Reward

Eating behavior is driven by the need to maintain energy homeostasis, which is critical for survival and regulated by central and peripheral systems. Food is the most potent natural reward conserved across species. An organism must remember where, when, and how to obtain food to continue survival and improve reproduction, particularly during times of scarcity. Homeostatic and hedonic mechanisms may interact to regulate food intake, especially when food is abundant, since certain foods are more or less “rewarding.” For example, palatable food has higher hedonic value than bland food and promotes greater activation within reward areas of the brain (see van der [27]). This activation closely parallels the activation pattern of drugs of abuse. Both drugs of abuse and food are potent reinforcers, which enhance dopamine release in reward-related brain regions (reviewed in [28]). Regardless of classification, rewards are powerful entraining forces, which can shift circadian rhythms.

### 2.1. Circadian Rhythms and Food

Beyond the hedonic value of food, organisms may use food and other stimuli to entrain, or adapt, to their environment to enhance survival; for example, the presence and consumption of food can shift circadian rhythms. Animals predominately eat during their active phase, with rhythms driving, seeking and feeding behavior at certain times of day. These feeding rhythms are in part regulated by circadian genes. Mutations in the *Per1* gene phase advances food intake into the sleep period, leading to obesity [29]. Similarly, *mPer2* knockout mice become obese after consuming food equally across the day and night (inactive and active phase, resp.) [30]. Day-night patterns of food intake are also abnormal in *ClockΔ19* mutants and BMAL1 null mice, with intake occurring more evenly across the 24-hour period [31, 32]. Moreover, metabolic homeostasis requires normal feeding rhythms, as shown by increased adiposity in rodents restricted to sleep phase feeding [33, 34]. On the other hand, active phase restriction of feeding prevents increased adiposity, glucose intolerance, and other measures typically induced by 24-hour access to high-fat diet [35].

Circadian rhythms are sensitive to changes in environmental stimuli, phase shifting (i.e., phase advance is the onset of the rhythm to an earlier time of day, while phase delay is the onset of the rhythm to a later time of day), and entraining across species to allow animals to adapt their behavior when necessary. Food is a potent stimulus of rhythm entrainment. Regardless of caloric restriction, locomotor rhythms will shift toward the time of a daily meal or a highly palatable snack (reviewed in [36]). For example, mice will begin to shift their activity into the light (inactive) phase, representing an advance of locomotor rhythms, when given chocolate without food restriction repeatedly during midday [37]. Furthermore, restricting feeding to a 12-hour period shifts the phase of circadian gene expression in peripheral cells, but not the SCN [38].

In addition to entraining circadian oscillators, food can reorganize temporal activity, for example, diurnality can be induced in rodents by a timed, daily palatable meal [37] or working for food while food restricted [39]. However, when mice are required to work to obtain chocolate with access to *ad libitum* chow, no shift in activity is observed [37]. This suggests that animals will continue to seek out food during their active phase, even when palatability is high if they are not energetically challenged. More importantly, these results indicate that an animal's metabolic state and the palatability of food play critical roles in how food shifts circadian rhythms. The shift seen in animals working for food seems to require an energetic challenge since it cannot be induced by increased running wheel activity or increased reward or palatability [37]. Furthermore, a daytime food reward is sufficient to induce a shift to diurnality even though this shift is smaller than that observed in the presence of food restriction ([39]; van der [40]).

Since metabolic state is important for inducing robust shifts in behavioral circadian rhythms, it is a fairly common practice to utilize food restriction [38, 41, 42]. However, when attempting to measure how food shifts and entrains rhythms in brain activity, the use of food restriction may be problematic. Hunger induced by food restriction could mask or artificially inflate the effects of a meal; therefore, it is important to include an *ad libitum* fed control group. Alternatively, a timed exposure to palatable food can be used alongside *ad libitum* access to chow. For example, a modest amount of entrainment in c-Fos expression occurs in the hypothalamus, which controls hunger, when a palatable food is present [43].

In addition to food entraining circadian rhythms, animals will learn to associate environmental cues, such as time of day with a food reward, or a scheduled meal. It is critical to account for time of day when studying food-related behaviors. The time of training and testing must overlap and be restricted to a certain circadian timeframe when studying conditioned place preference for food. Here, time is an internal circadian contextual cue that is critical for food-related behaviors in certain rat strains as well as marmosets [44, 45].

Anticipation, which is observed before a scheduled meal, particularly in rodents, is another example of a strong association between time of day and food. An increase in locomotion, core body temperature, and corticosterone usually precedes a timed meal (reviewed in [26]). The SCN does not appear to play a role in food entrainment since lesions fail to prevent the formation of anticipatory rhythms associated with restricted feeding schedules [46]. Although the brain regions responsible are largely unknown, the food-entrainable oscillator may be neuronal since anticipatory activity is resistant to adrenalectomy [47, 48] and cirrhosis [49]. Discovering the brain region, or regions, responsible for anticipation has been challenging. The food-entrainable oscillator could consist of a neuronal network, or circuit, and not a singular brain region [50], which is further complicated by the implication that the involvement of an oscillator could vary depending on the type of food.

In general, palatable food produces distinct effects compared to standard lab chow. Palatable food, such as chocolate, increases c-Fos expression, or activation, of corticolimbic regions including the prefrontal cortex (PFC) and nucleus accumbens (NAc) [51]. Moreover, the addition of palatable sucrose activates distinct neuronal networks in chow-restricted mice. Chow restriction entrains multiple subregions of the hypothalamus, activating the lateral, dorsomedial, and paraventricular nuclei during anticipation [42, 52]. The addition of palatable sucrose displaces most hypothalamic activity induced by restricted feeding, instead of activating the PFC, lateral septum, and NAc [42]. Relative to mice with restricted access to standard chow, mice with access to chocolate display a greater increase in amplitude and larger phase shift in rhythm of PER1 in corticolimbic structures, such as the amygdala, NAc, and PFC [41]. On the other hand, food restriction induces a higher amplitude of PER1 in the hypothalamus than chocolate [41]. These findings suggest that the mechanisms underlying anticipation of food may be distinct, depending on the type of food, and involve molecular rhythms.

**Figure 1:**
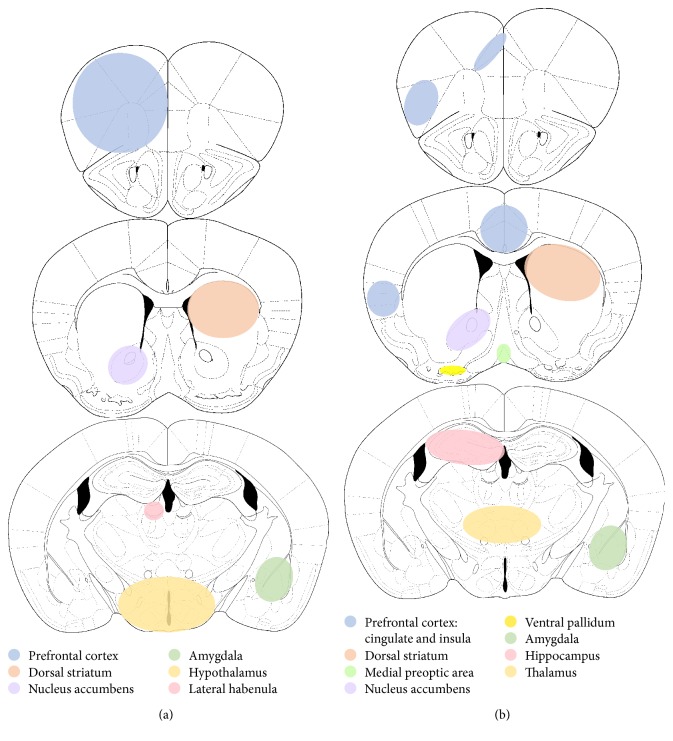
Multiple brain regions are activated or regulated by reward. Anticipation of (a) natural rewards, namely, food and (b) drugs of abuse activate discrete circuitry. The rhythmic expression of circadian genes in this and other brain regions is also altered by food or drugs of abuse demonstrating one potential mechanism by which reward influences circadian rhythms. (a) Anticipating a scheduled meal or palatable snack activates or increases c-Fos expression in the prefrontal cortex (PFC), nucleus accumbens (NAc), central amygdala, and hypothalamus. Furthermore, palatable food alters the expression of circadian genes in these and other brain regions. *Per1* rhythms are altered and shifted in the amygdala, NAc, and PFC, whereas the amplitude of *Per2* rhythms are intensified in the lateral habenula [116] as well as the suprachiasmatic nucleus (SCN not shown), cortex, and striatum [117] during anticipation of chocolate or after consumption of a high fat/high sugar diet or sucrose, respectively. (b) Anticipating drugs of abuse can activate multiple brain regions including the caudate, thalamus, insula, NAc, hippocampus, ventral pallidum, and cingulate. Similar to food, drugs of abuse also alter the expression of circadian genes. Specifically, cocaine alters the rhythm of *Npas2* and *Drd3* as well as the expression of numerous circadian genes in the NAc, most of which are upregulated. Circadian rhythms of period genes are also altered after withdrawal from morphine in the SCN, PFC, NAc, central and basolateral amygdala, hippocampus, and ventral tegmental area (not shown). Although it is beyond the scope of this figure to examine the differences in activation and circadian gene expression in various stages of addiction, it is important to note that acute versus chronic exposure to rewards have some distinct effects on the reward circuity in the brain. For example, it is known that the role of various brain regions shifts during extended exposure to drugs of abuse (reviewed in [118]). It is thought that this shift contributes to the transition from use to abuse to dependence. Similarly, circadian genes may be contributing to this transition since mutations in these genes cause a loss of rhythmicity [16], which is also thought to contribute to loss of control over drug taking and the transition to addiction.

Food-anticipatory activity is likely driven by both metabolic needs and reward. Accordingly, anticipation of palatable food entrains and/or activates the hypothalamus, even in the absence of food restriction, as well as reward-related brain circuitry (see Figure 1). Daily access to a palatable, fatty meal induces a modest amount of entrainment in c-Fos expression in the hypothalamus [43] and the anticipation of chocolate is associated with increased entrainment of PER1 expression in the dorsomedial hypothalamus [41]. Reward, motivation, or “wanting” also appear to contribute to anticipatory activity when limited access is given to a palatable food. For example, c-Fos expression is also induced in many corticolimbic structures, such as the PFC, NAc, and central amygdala [52].

Importantly, while ingesting, palatable food induces activation in reward-related brain regions such as the PFC and NAc and repeated presentations are required for these regions to become activated *during* anticipation [51]. Since repeated presentations are also required for anticipation to occur, anticipation-induced corticolimbic activation could underlie anticipatory activity. Furthermore, reward-related activation predating anticipation-related activation suggests that while the circuits mediating reward and anticipation may not be completely distinct, the underlying mechanisms may be different, but further study is needed. The transition from reward-induced activation to anticipation-induced activation in the case of food may extend to other rewarding substances, such as drugs of abuse, and importantly, could be a major contributor to relapse.

### 2.2. Potential Peripheral Mechanisms of Reward

In addition to the central mechanisms underlying reward and reward anticipation, peripheral oscillators may also impact reward. Primarily, peripheral hormones like estradiol and cortisol (corticosterone in the rodent) modulate neuronal activity, in turn altering circadian activity and reward. These peripheral hormones cycle in a daily rhythm. For example, estradiol cycles in an ultradian rhythm repeating approximately every 6 hours with an asymmetric peak in the early morning in humans [53]. Estrogen is known to modulate activity in circadian brain regions like the SCN where 17beta-estradiol administration increases firing frequency and miniature excitatory postsynaptic currents. This suggests that estrogen could be modulating and regulating circadian rhythms [54].

Fluctuations in circulating gonadal hormones, through treatment or normal menstrual cycling, also alter reward-related activity in the brain. In humans, when estrogen is unopposed by progesterone during the follicular phase of menses, reward-related brain regions are more active during anticipation, including the orbitofrontal cortex, amygdala, and striatum [55]. Hormone treatment early in menopause also increases anticipatory activity in the reward system, specifically the striatum and ventromedial PFC [56]. In nonhuman primates and rodents, the effects of estradiol on reward-related behavior are mixed. The reinforcing effects of cocaine do not vary across menstrual cycle phase in rhesus macaques [57], whereas estradiol treatment increases cocaine intake [58] and enhances acquisition for self-administration in rodents [59], while concurrent progesterone administration inhibits these effects. Together, these results suggest that estradiol plays an important role in reward, although some species differences exist. Generally, higher estradiol levels, whether through natural cycling or administration, promote reward-related behaviors as well as activity in reward-related brain regions.

Glucocorticoids, or stress hormones, which are released when the hypothalamic-pituitary-adrenal axis is activated by stress, also play a role in reward across species, but the results are mixed. These hormones also cycle over the 24-hour period, with a peak in the early morning during the daily shift to activity. In rodents, decreased glucocorticoids through adrenalectomy decrease reward through effects in the NAc, namely, decreased extracellular dopamine and Fos expression ([60]; reviewed in [61]). In humans, when high doses of cortisol are administered, activity in the striatum and basolateral amygdala decreases, but this is not specific to reward condition in a monetary incentive delay task [62]. On the other hand, acute stress, which can increase cortisol, decreases activity in the dorsal striatum and orbitofrontal cortex during reward [63]. These results demonstrate that the effects of glucocorticoids on reward are still unclear. Species differences alone do not drive these mixed results, since the method of glucocorticoid induction (whether stress-induced or through exogenous administration) also appears to play an important role.

Together, it appears that peripheral hormones may play a key role in modulating reward-related brain regions and may bridge the gap between related changes in circadian rhythms and reward. Future research should focus on the importance of the oscillations of these peripheral hormones on reward since it is still unknown as to whether the circadian nature of these hormones is relevant to their roles in reward.

### 2.3. Neural Mechanisms of Food and Drug Reward

The long-standing question of whether any reward, whether “natural,” such as food or sex, or drugs of abuse, has the same underlying mechanisms still remains largely unanswered. The brain evolved to respond to natural rewards, which are necessary for survival and reproduction. Both uncontrolled food intake, which is often compared to drug addiction, and drugs of abuse “hijack” reward and motivation associated neural circuitry (reviewed in [64]). Across species, both natural rewards and drugs of abuse predominately affect the corticolimbic reward system.

Palatable food, like drugs of abuse, has reinforcing effects mediated in part by enhanced dopamine release into reward-related areas of the brain (reviewed in [28]). For example, both sucrose and saccharin increase extracellular dopamine in the NAc of rats [65, 66], while palatability of food correlates with dopamine levels within the dorsal striatum [67]. Moreover, milkshakes, a highly palatable food, trigger striatal activation, whereas frequent ice cream consumption blunts striatal responses in humans [68]. The activation of reward circuits by repeated, restricted access to food, as discussed prior, can induce anticipatory activity. Similarly, exposure to drugs of abuse, such as methamphetamine and cocaine, induces anticipatory activity, potentially through entrainment of the circadian clock [23–25].

Although both food and drugs of abuse entrain rhythms, a single oscillator may not be responsible for generating reward-entrained rhythms. Rats are capable of entraining and showing anticipation, to both food and cocaine rewards separately, indicating that rewarding stimuli can be differentiated in a circadian fashion. Moreover, two separate free-running rhythms occurred after entrainment to food and cocaine rewards ceased. Together, these findings suggest that more than one oscillator generates reward-induced rhythms [23].

In addition to differences in circadian entrainment, anticipation of both food and drugs of abuse recruit corticolimbic brain regions (see Figure 1). Anticipation of nicotine due to the presentation of cues activates the caudate, thalamus, insula, NAc, and cingulate in humans [69]. In rats, anticipation of ethanol after the presentation of cues increases activity in the caudate, insula, hippocampus, ventral pallidum, NAc, and medial preoptic area [70]. This brain activity appears to be due to building anticipation induced by cues since repeated presentations over 5-6 days are required. This is similar to PFC and NAc activation induced by food anticipation, which requires 3–5 repeated presentations [51]. Although these studies did not use circadian entrainment of drug anticipation, they demonstrate that similar prefrontal cortical and striatal brain regions are active during anticipation of both food and drugs of abuse.

Interestingly, the hippocampus, medial preoptic area, and dorsal striatum appear to be activated by drug anticipation alone. These regions may have simply not been studied in food anticipatory experiments or these may be truly distinct effects. Future studies will need to examine additional brain regions of interest in order to determine which regions are universal, and which play a role in drug anticipation only. Another important consideration is that most experiments measuring brain activation induced by food anticipation investigate the PFC as one brain region [51, 52]. In future studies, it will be critical that distinct PFC subregions, such as the cingulate, orbitofrontal, prelimbic, and insula are examined separately. These various subregions of the PFC should be studied independently since they are critical for different features of addiction. Furthermore, this more specific analysis will allow for a better, more in-depth comparison of results between species and will paint a clearer picture of the roles of cortical subregions identified in human drug anticipation experiments, such as the insular and cingulate cortices.

On the other hand, the amygdala and the hypothalamus were only activated by food anticipation, even though both regions are believed to contribute to drug reward. The amygdala is critically important for the drug-related learning critical for associating cues with drugs of abuse [71], and studies suggest that the hypothalamus is involved in drug seeking. For example, hypothalamic orexin/hypocretin neurons play a role in cocaine seeking but do not appear to contribute to seeking motivated by a palatable food [72]. These results suggest that even though the hypothalamus is involved in metabolic regulation, food ingestion, and anticipatory activity in food-entrained rodents, distinct mechanisms likely underlie reward seeking between food and drugs of abuse. Taken together with findings that food and subsequently anticipation alone activate reward-related brain regions, these results suggest that although the brain regions mediating reward for food and drugs of abuse may be similar, how/when the regions are recruited and the underlying mechanisms by which those regions regulate reward may be very distinct.

In addition to parallels in brain circuitry, there are other fundamental differences in food and drug reward mechanisms. Palatable food is sometimes preferred over drugs of abuse, for example, rats tend to prefer saccharin and sucrose to cocaine [73]. Moreover, it has been shown that half of the mice tested prefer peanut butter compared to peanut butter mixed with methamphetamine [24]. From an evolutionary perspective, it is reasonable for food to be highly potent and rewarding since it is critical to survival. Since self-administration of highly palatable food, compared to drugs of abuse, is less time-consuming and complex, we should continue to leverage the potency of food to study the underlying processes giving rise to reward versus anticipation as well as increased and uncontrollable food intake. Despite food being a more potent reward in some circumstances, drugs of abuse have greater consequences in other regards. For example, associations between drugs of abuse and stimuli last longer than for natural reinforcers, indicating cues are especially powerful in the context of drug use (discussed in [64, 74]).

In the future, studies should continue to examine different types of reward in parallel. This will allow us to elucidate whether the mechanisms underlying the rewarding effects of drugs of abuse and natural rewards are different. Identifying the parallels and distinctions across types of reward could help us treat both obesity and drug addiction.

## 3. Loss of Rhythmicity and Loss of Control

### 3.1. Drugs of Abuse Alter Rhythms

Like natural rewards, there are diurnal variations in behavioral responses to drugs of abuse and drug taking in self-administration paradigms [19, 75]. For example, drug intake and self-administration of cocaine and ethanol are higher in rodents at night, during the active phase [19, 20, 76]. Not only are there natural rhythms to drug taking and reward, but exposure to drugs of abuse induces behavioral, anticipatory rhythms, potentially through entrainment of the circadian clock ([23–25]; reviewed in [77]).

Drugs of abuse induce behavioral rhythms, but drug exposure is also known to regulate circadian genes and alter circadian rhythms. Exposure to drugs of abuse alters expression of circadian genes, such as, *Period*, *Clock*, and *Npas2* ([15, 78]; reviewed in [77]). More importantly, volitional cocaine intake via self-administration regulates circadian genes, upregulating *Clock*, *Bmal1*, *cryptochrome1* (*Cry1*), and *Period2* (*Per2*) [20]. In addition, cocaine can induce phase shifts in circadian rhythms (reviewed in [77]). For example, even one acute injection of cocaine causes mice to phase advance 1 hour, and this effect is exacerbated in *Per2* mutant mice. These findings suggest that cocaine might also regulate free-running circadian rhythms.

### 3.2. Loss of Rhythmicity in Drug Self-Administration

Chronic exposure to drugs of abuse also causes a loss of rhythmicity in locomotor activity. These altered diurnal activity patterns are usually driven by increases in locomotor activity during the inactive period. For example, repeated amphetamine administration increases daytime locomotor activity in adolescent female rats [17]. Chronic alcohol exposure also increases daytime locomotor activity in mice, eliminating the typical rhythm as well as the peak in activity during the early night [79]. Over time, drugs of abuse also cause a loss of rhythmicity in self-administration and drug intake. Extended access or access to high doses of cocaine causes a loss of rhythmicity in drug taking, as evidenced by responding that continues well into the light phase, in lieu of predominating at night [19].

Drug exposure can alter rhythmicity, but mutations in circadian genes can also impact self-administration. Mice with a dominant negative mutation in the Clock gene self-administer cocaine equitably during the day and night. On the other hand, wild-type mice fail to acquire criteria for self-administration, but only during the day. This suggests that decreased Clock function increases vulnerability for cocaine use, at least in part by reducing rhythmicity and increasing drug use during the day [16]. Deletion of the circadian rhythm gene *Per1* also causes a loss of circadian rhythmicity in alcohol intake, again normalizing intake across time of day [80]. Loss of rhythmicity can also be seen for natural rewards, like palatable food. *mPer2* knockout mice eat the same amount of a high-fat diet during the day and night, which leads to obesity [30].

In addition to a loss of rhythmicity, chronic exposure to drugs of abuse can also cause an overall increase in, or loss of control over, drug intake. Increases in drug taking can be seen across species in extended access paradigms. In rodents, extended access to cocaine causes escalation and the development of excessive drug intake [81, 82]. In nonhuman primates, unlimited access produces erratic and rapid binge-like patterns of cocaine self-administration, which can ultimately lead to seizures and death [83, 84]. Escalated or excessive drug intake and as compulsive or habit-like drug seeking despite adverse consequences are primary features of drug addiction, which contribute to the development of substance dependence. Therefore, a loss of control over intake could model a transition to an addiction-like state.

Loss of control or escalation in drug taking appears to be closely paralleled by, and potentially driven by, a loss of rhythmicity and increased daytime responding in rodents [19]. When drug taking increases during the inactive phase, it reflects a loss of control in drug seeking and taking. In this case, animals are likely sacrificing time usually dedicated to sleep and rest. However, it is difficult to propose a causal or correlational relationship between loss of rhythmicity and loss of control because only a few key studies have examined rhythmicity or patterns of drug taking across a 24-hour period [19, 80]. Others, that do examine phase differences, do not always compare initial drug use to use after long-term exposure. Therefore, even if no differences in day-and-night responding are found, this cannot be correlated to an escalation of intake over time [85].

These questions are understudied because they are especially difficult and time-consuming experiments. Due to cost and space limitations, it is difficult to study self-administration over a 24-hour cycle. In addition to experimental challenges, unlimited access to drugs of abuse can cause death or health deterioration in animals. An alternative method is to use discrete trials spread out over a 24-hour period [19] or to use shorter access periods of self-administration and compare animals that self-administered during either the active or inactive phase.

In addition to being a key feature of addiction, escalation and loss of control over drug taking also contribute to other addiction-related behaviors. For example, paradigms which produce escalation also increase reinstatement of drug seeking, a model of relapse [86]. On the other hand, the role of rhythmicity in other addiction-related behaviors is less clear. In order to determine whether rhythmicity might play a role in other addiction-related behaviors, rhythms of self-administration can be correlated with measurements of reinforcement, motivation, and/or relapse-like behavior. If altered rhythmicity plays a critical role in the progression to substance dependence, it should contribute or at least correlate to other critical behaviors related to the overall phenotype of addiction.

In order to study the relationship between rhythmicity and loss of control, it is important to consider which parameters trigger escalation and compare those to parameters which do not: more versus fewer discrete trials, high versus low unit doses, and longer access sessions (approximately 6 versus 1 hour). Studies might successfully investigate the link between loss of rhythmicity and loss of control if these parameters are investigated further. For example, if we can slow down the progression of escalation, we can determine whether a loss of rhythmicity occurs prior to and is therefore potentially contributing to escalation.

Overall, parallels in loss of rhythmicity and loss of control have been difficult to connect. Future research should strive to complete and more elegant long-term studies examining patterns of self-administration. Future experiments should build on the existing literature and examine how other factors, such as mutations in circadian genes, previous drug or stressor exposure, or developmental stage, affect patterns and timing of self-administration. At the least, studies should attempt to investigate self-administration during both the active and inactive phases. It is possible that these phases will shed light onto the mechanisms underlying the development of different phases of addiction, with dark phase self-administration paralleling the development of substance use/abuse, and drug taking during the light cycle paralleling loss of control and the development of substance abuse/dependence.

### 3.3. Sleep Dysregulation and Circadian Rhythms

One parallel between loss of rhythmicity and loss of control in drug use is sleep deprivation. Increased drug intake during the inactive period is probably interrupting sleep, and excessive drug intake causes animals to neglect sleep. For example, a 24-hour access to cocaine causes rapid, erratic, and continuous self-administration across phase, which continues until the point of exhaustion in nonhuman primates, when drug taking is discontinued for a period of sleeping and eating [83]. Even in rats, self-administration of cocaine can cause generally deteriorated health and increased mortality [87].

Drug exposure also has direct effects on sleep across species. Cocaine self-administration during the day decreases sleep efficiency and increases sleep fragmentation in rhesus macaques [22]. This is very closely paralleled in rats, where withdrawal from cocaine self-administration reduces nonrapid-eye-movement (NREM) and REM sleep and increases sleep fragmentation [21]. In addition, withdrawal from long access cocaine self-administration reduces glucose utilization in both reward-related (caudate, NAc, amygdala, etc.) and sleep-related (dorsal raphe, locus coeruleus, and thalamus) brain regions in rats [82]. These findings can also be extended to other drugs of abuse. After ethanol withdrawal, mice show disruptions in sleep, with reduced NREM sleep and increased REM sleep [88]. These results parallel human findings, where addicts report sleep disturbances [7, 89, 90]. More specifically, a reduction in sleep and an increase in sleep fragmentation are reported during cocaine withdrawal ([91]; reviewed in [6, 92, 93]).

These symptoms closely resemble chronic insomnia, and it is thought that these sleep disruptions promote relapse. Recent studies have investigated the role of cocaine-induced sleep fragmentation in cocaine craving by augmenting or attenuating REM sleep fragmentation following cocaine self-administration in rats. Interestingly, as REM sleep fragmentation changes, the incubation of cocaine craving changes in parallel [21]. In addition, chronic sleep deprivation increased drug taking and incentive motivation for cocaine in high drug-taking rats [94]. Together, these findings demonstrate the cyclical relationship between drug exposure and sleep, with drug use affecting sleep and impaired sleep further perpetuating drug use.

It is important to note that although these studies examine sleep, they do not necessarily investigate circadian rhythms. Sleep and circadian rhythms are often not distinguished, being discussed interchangeably. It is important that studies attempt to measure both sleep and circadian phenotypes in order to gain insight about where sleep and circadian rhythms converge and diverge. Circadian rhythms and sleep can be studied concurrently by measuring sleep through electroencephalography (EEG) and electromyography (EMG) as well as circadian rhythms of locomotor activity, body temperature, and so forth [95, 96]. PiezoSleep mouse behavioral tracking systems have made this easier since they measure sleep/wake cycles through floor sensors, eliminating the EEG-/EMG-associated surgery. In addition, sleep/wake rhythms can be measured *in vivo* with circadian oscillations in gene expression or protein levels being examined in tissue ex vivo [95, 96].

It is particularly critical to investigate sleep and circadian rhythm disruptions in addiction research since sleep disturbances are a major symptom of addiction, which can trigger relapse. Using sleep boxes or measuring circadian rhythms, ex vivo are particularly important for studies examining addiction-related behaviors where limiting additional surgical exposures is critical. For example, mice self-administering drugs of abuse have already undergone an intravenous catheterization, and in many cases, an additional viral vector placement. If an additional surgery for measuring EEG/EMG is not absolutely required, it should be avoided. Furthermore, measuring activity rhythms with wheel running should be avoided because of the risk that the exteriorized self-administration port would catch on the wheel and injure the animal. Regardless, it is difficult to measure sleep and activity rhythms *during* active self-administration since mice typically self-administer drugs in an operant chamber. However, these measurements can be taken for a period before and/or after the length of drug exposure or daily during the drug self-administration paradigm if the hours of self-administration, when mice are out of the home cage, are excluded. Sleep disturbances in addicts might be more effectively treated once we better understand what causes them; understanding circadian rhythms might shed light onto how drugs of abuse alter sleep.

### 3.4. Rhythms of Drug Self-Administration and Treatment

Rhythms of drug self-administration will also be critical to study independently of escalation. Importantly, self-administration of cocaine under 3 discrete trials/hour (one infusion available during each trial) is tightly coupled during the dark phase of a normal light/dark cycle. In constant, dim light self-administration continues to be rhythmic in the absence of light-dark cues, although it is progressively phase delayed. On the other hand, in constant light, rhythms of self-administration become fragmented and animals take significantly less cocaine [97]. Taken together, these results suggest that cocaine intake is produced through a circadian mechanism and that circadian control of self-administration is influenced by light.

Determining the mechanisms by which various factors affect the rhythm of drug self-administration, and drug intake overall, will inform future treatment options. In the future, studies should continue to attempt to limit not only drug taking as above but also motivation and drug seeking with changes to light conditions, including lengthened or shortened light exposure. This paper suggests that light (or darkness in diurnal species) could be a useful therapy to suppress cocaine intake. In addition to changes in light/dark exposure, the appropriate phase-controlled timing of additional treatments, perhaps during the times of day with the highest periods of intake, could be critically important to the efficacy of treating addiction.

## 4. Circadian Rhythms and Drugs of Abuse: Human and Animal Parallels

### 4.1. Circadian Clock Genes

Drawing parallels between human and animal methods, mechanisms, and treatments is one of the most challenging necessities facing research today. Research focused on circadian genes, and drug use has been making strides in this vein, as indicated by a fairly large literature paralleling the role of circadian clock genes in addiction in both rodents and humans. Two of the genes that have been most extensively studied are *Period* and *Clock.*

Mutations in *Per1* and *Per2* have been extensively studied in rodents. For example, *mPer1* and *mPer2* null mutant mice increase ethanol intake and conditioned place preference [14]. In humans, various single nucleotide polymorphisms (SNP) within Per1 and Per2 are associated with alcohol drinking. For example, one SNP in Per1 is associated with alcohol dependence [13]. Per1 is also associated with drinking and stressor exposure. *mPer1* mice consumed more alcohol in response to social defeat stress [13], whereas a SNP in Per1 interacts with early life stress to predict problematic alcohol use in humans [8]. Two different SNPs in Per2 are associated with stressful events and alcohol drinking in young adults [10] as well as reward dysfunction in humans, respectively [11].

Mutations and variants in *Clock* are also associated with drug use across species. In rodents, *ClockΔ19* mice, with a dominant negative mutation in the Clock gene, have increased ethanol intake and self-administration of cocaine [15, 16]. In humans, variants in the CLOCK gene are associated with heavy cocaine use, although not DSM-IV diagnosis of dependence [9].

Even though there is extensive research on circadian rhythm genes and drug use across species, it is unclear whether these findings tell us something important. For example, on a mechanistic level, it is uncertain whether SNPs matter. The research is the most complete for alcohol, but SNPs have not been studied systematically across drugs of abuse. Furthermore, SNPs do not seem to always correlate with the severity of drug use and dependence. It is also unclear whether the mechanisms underlying the association between SNPs and drug use in humans actually contribute to the role of circadian genes in addiction. More technologies and mouse models are being created so we are now more capable of paralleling findings in humans and animals. With the advent of CRISPR, it is much easier to create humanized mouse lines with targeted changes to specific genes. Future studies should aim to identify whether the effects of SNPs associated with drug use or dependence in humans parallel the effects of mutations in *Period* or *Clock* genes in rodents. If effects are consistent across mutations and SNPs, that would suggest the mechanisms underlying these effects are similar and the parallel between human and rodent literature is stronger. These mouse lines can then be used to probe for underlying mechanisms and develop potential therapeutics based on those mechanisms and then the clinical researchers can build on these mechanisms to confirm they are paralleled in humans or test novel therapies.

### 4.2. Dopamine Receptors in the Striatum

Drugs of abuse, specifically psychostimulants, activate dopamine signaling, overriding natural reward circuitry. The ratio of D1 and D2 receptors plays an important role in the rewarding effects of cocaine; D1R signaling appears to enhance [98], while D2R signaling decreases cocaine reward [99]. In addition, future cocaine preference negatively correlates with D2-/D3R-binding availability in the ventral striatum in rats [100]. These results indicate that a predominance of D1R over D2R signaling must be present for the rewarding effects of cocaine. It has also been hypothesized that the transition to compulsive intake could result from this predominance.

In mice, acute cocaine causes both fast stimulation of D1R and slower stimulation of D2R containing neurons in the dorsal striatum [101]. This suggests that cocaine reward may be mediated by not only the abrupt activation of D1R neurons but also a longer-lasting, progressive deactivation of D2R neurons in the dorsal striatum [101]. Interestingly, in mice exposed to chronic cocaine, acute cocaine stimulates D1R and D2R neurons less, while inducing a slower, yet longer-lasting increase in D1R activation. This altered D1R neuron stimulation induced by acute cocaine extends the predominance of D1R over D2R signaling, which is typically rapid and short lasting [102]. Striatal D2R downregulation might contribute to the sustained signaling imbalance.

Similarly, cocaine abuse potently affects dopamine receptors in the striatum in humans, reducing D2/3 availability, which is associated with increased drug craving [103] and vulnerability to relapse [104]. Cocaine abusers show dampened stimulant-induced increases in dopamine, as shown by reduced D2/D3 receptor binding, in the striatum, despite the induction of intense craving [105]. Interestingly, reduced D2R signaling during intoxication is maintained even when cocaine-associated cues are present [106]. It has been hypothesized that this reduction in D2 signaling could be contributing to compulsive drug use.

**Figure 2:**
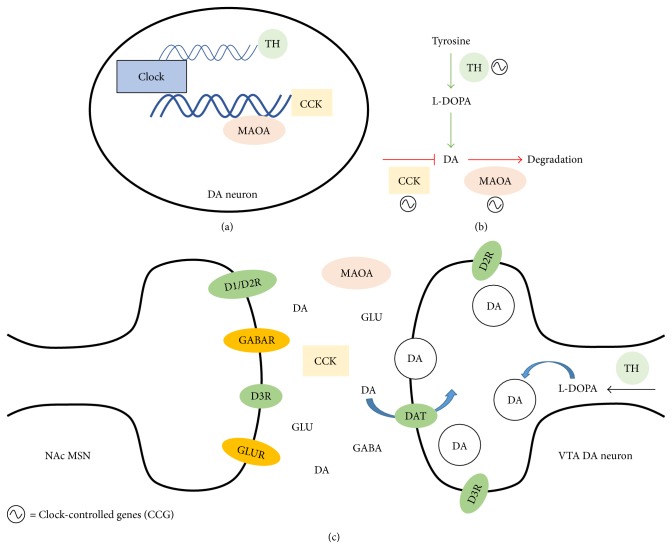
Potential mechanisms by which circadian rhythms impact reward. One of the primary mechanisms by which circadian rhythms might affect reward and reward-related behaviors is through monoaminergic signaling in the nucleus accumbens (NAc). In particular, circadian clock genes control regulatory processes involved in the synthesis, function, and degradation of dopamine (DA). Clock is thought to be a negative regulator of reward, since mutations in the *Clock* gene increase preference and drug taking. CLOCK and other related genes may be negatively regulating DA through their transcriptional actions. (a) Overall, dopaminergic signaling is reduced through CLOCK's actions (and increased in *ClockΔ19* mutant mice) because genes that increase dopaminergic signaling are negatively regulated by clock genes while genes that decrease dopaminergic signaling are positively regulated. (b) Tyrosine hydroxylase (TH), the rate-limiting enzyme in the synthesis of dopamine (DA), is negatively regulated, whereas monoamine oxidase A (MAOA), an enzyme which breaks down DA in the synapse and cholecystokinin (CCK), a regulatory peptide which negatively affects DA output is positively regulated by clock genes [114, 119]. (c) These changes in dopaminergic neurons in the VTA influence dopaminergic, and potentially glutamatergic, signaling in the NAc, which could mediate increases in reward seen in mice with mutations in clock genes. This could hold true in humans, with circadian disruptions or polymorphisms in circadian genes increasing dopaminergic signaling in the NAc, which leads to increased vulnerability to substance dependence.

Dopamine may also play a critical role in the relationship between reward and circadian rhythms (see Figure 2). Extracellular dopamine fluctuates rhythmically in the dorsal striatum, with its peak amplitude during the active phase [107]. Furthermore, D2R activity contributes to the rhythm of *Per2* expression in striatum [107], indicating that dopamine interacts with the circadian clock in reward-related brain regions. Therefore, drug-induced changes to the dopamine system may play a critical role in the disruption of circadian rhythms in addicts. For example, sleep disturbances are one of the key effects of chronic drug use in rodents [82, 87, 88] and in humans [7, 89, 90].

Sleep-wake disruptions may be regulated not only by circadian rhythms but also by ultradian rhythms, which are cycles that repeat every few hours in the body, such as alertness and appetite. The dopaminergic ultradian oscillator cycles with the circadian clock, but the two can become desynchronized when dopaminergic tone is elevated like in drug abuse [108]. This desynchronization can lead to aberrant arousal, similar to disturbed sleep-wake cycles seen across psychiatric disorders, including addiction. However, less is known about the mechanisms underlying ultradian rhythms compared to circadian rhythms. Future research should focus on dissecting the contributions of ultradian and circadian rhythm disturbances to drug addiction.

The relationship between circadian rhythms and addiction appears to be cyclical. Dopamine and drugs of abuse can disrupt rhythms, but sleep disturbances can also affect the dopaminergic system. Sleep deprivation decreases availability of D2 and D3R in the ventral striatum [109]. This finding seems to be a result of a downregulation of D2/D3 receptors in the ventral striatum, in lieu of dopamine increases, after sleep deprivation [110]. In this particularly translational study, a similar lack of change in dopamine levels in the ventral striatum was also seen in rats after one night of sleep deprivation [110]. Importantly, in cocaine abusers, sleep duration predicts D2/D3 availability [111]. This suggests that impaired sleep contributes to lower striatal D2/3 in cocaine abusers and that intervening with sleep patterns could improve treatment outcomes. However, it is again important to note that sleep disturbances might not necessarily correspond to changes in circadian rhythms.

Several studies have examined relationships between drugs of abuse, dopamine receptor type, the ventral striatum, and circadian genes. One study indicates that circadian genes might play a role in regulating dopamine receptor availability, possibly in the context of drug exposure. For example, striatal D2R levels are associated with the genotype of a novel variable number tandem repeat polymorphism in the *PER2* gene. Furthermore, genotype distribution is varied in cocaine abusers compared to controls [112].

Another study found that disturbing circadian rhythms with exposure to constant light increases expression of Per1, Per2, and D1R in the striatum could contribute to vulnerability to morphine consumption and preference in rats [113]. In mice, genetic manipulations of circadian clock genes impact cocaine preference as well as dopamine receptor expression (see Figure 2 for potential mechanisms). A dominant negative mutation in the *Clock* gene increases cocaine preference [114], whereas knocking out *NPAS2*, or knocking down NPAS2 in the ventral striatum, decreases cocaine preference [115]. *Clock*, and more so *Npas2*, is enriched in D1-containing neurons in the striatum, and ventral striatal NPAS2 knockdown disrupts the rhythmic expression of *Drd3* (dopamine receptor 3) [115]. Furthermore, chronic cocaine exposure disrupts diurnal rhythms in *Drd3* and *Drd1* [115].

Recently, a fair amount of research has investigated the role that circadian genes play in the effects of drugs of abuse on dopamine receptor availability and distribution in the ventral striatum. These studies emphasize the importance of circadian rhythms and circadian genes in one of the most prominent and well-studied mechanisms underlying addiction. Moving forward, we should continue to lay the groundwork for understanding how circadian rhythms and addiction are connected.

## 5. Summary

Taken together, these findings could help answer one of the most important questions facing the field of circadian rhythms and addiction today: do rhythms matter for treatment? Circadian rhythms appear to be important for drug taking and potentially for the transition to substance dependence and if rhythms matter for patient outcomes. It is critical to continue answering the questions posed in this review. Based on findings that sleep disturbances, whether induced by drugs of abuse or not, disrupt reward-related circuitry, it appears that treating sleep disturbances could be critically important. These treatments could occur preventively, for example, during development when sleep disruptions begin, or retrospectively, in the case of drug exposure where sleep disturbances are a symptom of use and withdrawal, which contribute to relapse. Moreover, determining the mechanisms by which drug taking is regulated by the circadian clock will lead to the development of novel therapies, including light therapy, and/or treatment approaches, such as phase-controlling treatment.

Fortunately, parallel findings studying circadian rhythms and/or sleep, and drug addiction are becoming more frequent in animal models. However, it is important to remember the shortcomings of mouse models. For example, the SCN appears to adapt much faster in rodents than it does in humans, which could lessen the severity or impact of sleep disruptions in rodent models. Therefore, care should be used when directly comparing circadian impairments and sleep disruptions across species.

Due to this additional factor, clinical research should take advantage of the mechanistic findings from animal models and try to create therapies for drug-induced impairments in circadian rhythm, but it is possible these mechanisms will be slightly different. Most of the animal literature is still building on a few, older human studies with very small sample size; therefore, updating and building on the existing human literature is imperative. In particular, studies aimed at manipulating circadian sleep and then investigating the effects on reward or vulnerability to drug use would be a crucial addition to the field.

Despite the difficulties, paralleling animal and human behavioral studies and manipulations will ensure the field progresses rapidly. Clinical research is critical, but we need to leverage those findings by using animal models to discover possible mechanisms and gain information about potential druggable targets. These results can be brought full circle into human studies in order to improve treatments options and approaches.
